# Traumatic funicular phlebitis of the thoracic wall resembling Mondor's disease: a case report

**DOI:** 10.1186/1752-1947-5-127

**Published:** 2011-03-30

**Authors:** Takeshi Kondo

**Affiliations:** 1 Division of Legal Medicine, Department of Community Medicine and Social Healthcare Science, Kobe University Graduate School of Medicine, 7-5-1 Kusunoki-cho, Chuo-ku, Kobe 650-0017, Japan

## Abstract

**Introduction:**

Mondor's disease is a peculiar form of thrombophlebitis, involving a superficial vein in the subcutaneous fat of the breast or anterior chest wall.

**Case presentation:**

The author presents a case of a 35-year-old male Japanese patient with cord-like induration in the right lateral thoracic wall. This lesion was diagnosed as traumatic funicular phlebitis, resembling Mondor's disease.

**Conclusion:**

Traumatic funicular phlebitis, resembling Mondor's disease, is a clinical entity which may give suggestive insight to the etiology of Mondor's disease itself.

## Introduction

Mondor's disease is a peculiar form of superficial thrombophlebitis, first reported in 1939 [1] as a thrombophlebitis involving a superficial vein in the subcutaneous fat of the breast or anterior chest wall, especially in women [2]. Classic Mondor's disease involves the lateral thoracic, thoracoepigastric, or superior epigastric veins [3]. It usually occurs as a sudden, subcutaneous tender, painless, cord-like swelling of the vein [2]. The process is usually unilateral, but very rarely bilateral manifestations have been found. The histologic changes are limited to a "subcutaneous vein showing thrombosis and organisation" [4]. This article will describe a case which can be called traumatic funicular phlebitis.

## Case presentation

**Figure 1:**
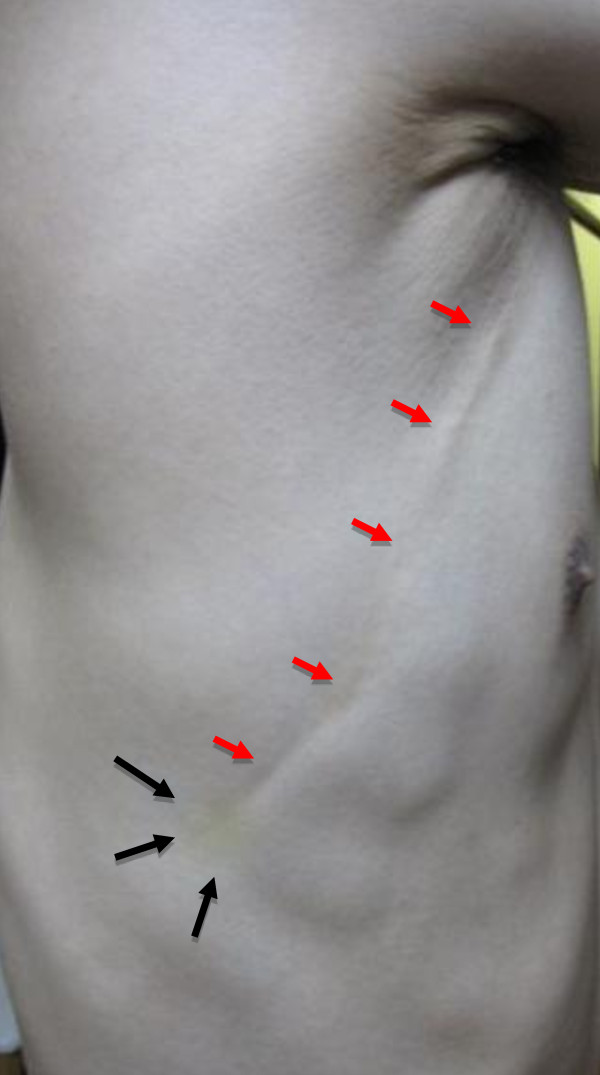
**Macroscopic findings of the lesion**. The lesion (red arrows) in the right thoracic wall was approximately 15 cm long, extending from the painful point with old (yellowish) subcutaneous hemorrhage (black arrows) to the axilla.

**Figure 2:**
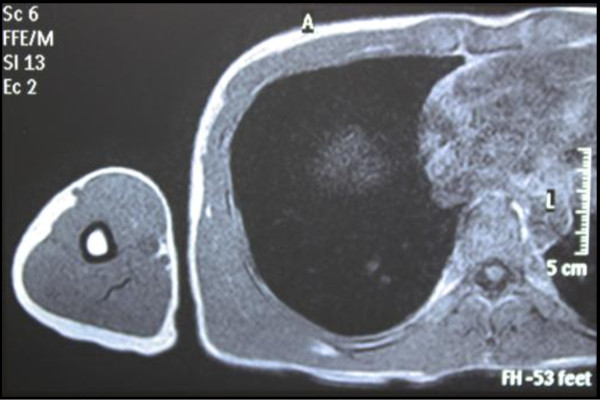
**MRI image (T1-weighted)**. MRI imaging detected no lesion in the right thoracic wall.

A 35-year-old Japanese man, working for a pathological laboratory, noticed tenderness in his right lower lateral thoracic wall and a palpable cord-like lesion extending from the painful point (on the right seventh rib) to the axillar fossa. Physical examination revealed the presence of a curvilinear subcutaneous cord-like induration in the right lateral chest wall. The lesion was approximately 15 cm long, originating from the painful position (on the right seventh rib) with rather old subcutaneous hemorrhage to the axillar fossa (Figure 1). The old subcutaneous hemorrhage suggested the traumatic origin, although the patient did not remember the traumatic event. Based on the color, consistency and shape of the hemorrhage (or ecchymosis), the possible traumatic event had occurred two or three weeks before. The funicular lesion anatomically corresponded to the right thoracodorsal vein. The overlying skin was freely mobile, and did not show any inflammatory signs. No other symptoms were reported. Magnetic resonance imaging (MRI) did not detect any lesion corresponding to the subcutaneous cord-like lesion (Figure 2).

Based on the above findings, the lesion was diagnosed as traumatic funicular phlebitis (resembling Mondor's disease) of the right thoracodorsal vein.

The lesion spontaneously regressed for approximately three weeks and, on follow-up, there have been no signs of recurrence for months.

## Discussion

The exact cause of Mondor's disease is still unclear, and its etiopathogenesis has been controversial. Various authors have associated the disease with local trauma, including biopsy or surgery, and muscular strain. Inflammatory and infectious agents have also been considered as etiologic factors [3,4]. Furthermore, Mondor's disease may herald an occult breast cancer [5]. Mondor's disease can be called Mondor's vasculitis (phlebitis or lymphangitis) [6], and this case can be called traumatic funicular phlebitis showing "Mondor-like" symptoms. This case can be called Mondor's disease, but the traumatic cause is not clear, so this lesion should be considered as a new entity: traumatic funicular phlebitis (TFP).

Although in this case a pathological specimen was not available, the lesion was clinically considered as phlebitis of the right thoracodorsal vein caused by a local trauma.

Penile Mondor's disease is a variant outside the thoracic area (or a different clinical entity) and its pathogenesis is better understood than that of classical Mondor's disease [7]. Although penile Mondor's disease may be a totally different clinical entity, pulsed and color Doppler sonographic findings and magnetic resonance angiography (MRA) findings of penile Mondor's disease have been reported [7,8]. In this case, an MRA was not available. MRI imaging detected no lesion, suggesting the lesion was at the healing stage. Histologically, in the healing stage, connective tissue proliferation took place in the vessel, resulting in the formation of a hard cord. Thus the lesion was indistinguishable from the surrounding tissue, although it was palpable.

## Conclusion

In conclusion, traumatic funicular phlebitis, resembling Mondor's disease, is a clinical entity, which may give suggestive insight to the etiology of Mondor's disease itself.

## Consent

Written informed consent was obtained from the patient for publication of this case report and accompanying images. A copy of the written consent is available for review by the Editor-in-Chief of this journal.

## Competing interests

The author declares that he has no competing interests.
